# Characterization of the Uncertainty of Divergence Time Estimation under Relaxed Molecular Clock Models Using Multiple Loci

**DOI:** 10.1093/sysbio/syu109

**Published:** 2014-12-11

**Authors:** Tianqi Zhu, Mario Dos Reis, Ziheng Yang

**Affiliations:** ^1^Beijing Institute of Genomics, Chinese Academy of Sciences, Beijing 100101, China ^2^Department of Genetics, Evolution and Environment, University College London, Darwin Building, Gower Street, London WC1E 6BT, UK

**Keywords:** Bayesian inference, divergence time, finite-sites theory, infinite-sites theory, posterior variance, relaxed clock

## Abstract

Genetic sequence data provide information about the distances between species or branch lengths in a phylogeny, but not about the absolute divergence times or the evolutionary rates directly. Bayesian methods for dating species divergences estimate times and rates by assigning priors on them. In particular, the prior on times (node ages on the phylogeny) incorporates information in the fossil record to calibrate the molecular tree. Because times and rates are confounded, our posterior time estimates will not approach point values even if an infinite amount of sequence data are used in the analysis. In a previous study we developed a finite-sites theory to characterize the uncertainty in Bayesian divergence time estimation in analysis of large but finite sequence data sets under a strict molecular clock. As most modern clock dating analyses use more than one locus and are conducted under relaxed clock models, here we extend the theory to the case of relaxed clock analysis of data from multiple loci (site partitions). Uncertainty in posterior time estimates is partitioned into three sources: Sampling errors in the estimates of branch lengths in the tree for each locus due to limited sequence length, variation of substitution rates among lineages and among loci, and uncertainty in fossil calibrations. Using a simple but analogous estimation problem involving the multivariate normal distribution, we predict that as the number of loci (L) goes to infinity, the variance in posterior time estimates decreases and approaches the infinite-data limit at the rate of 1/L, and the limit is independent of the number of sites in the sequence alignment. We then confirmed the predictions by using computer simulation on phylogenies of two or three species, and by analyzing a real genomic data set for six primate species. Our results suggest that with the fossil calibrations fixed, analyzing multiple loci or site partitions is the most effective way for improving the precision of posterior time estimation. However, even if a huge amount of sequence data is analyzed, considerable uncertainty will persist in time estimates.

Bayesian estimation of species divergence times under the clock and relaxed clock models incorporating uncertain fossil calibrations has attracted much attention lately. Several computer programs have been developed that implement different priors of rates and times and different strategies for incorporating fossil calibrations, such as multidivtime (Thorne et al. 1998; Kishino et al. 2001), mcmctree (Yang and Rannala 2006; Rannala and Yang 2007; Inoue et al. 2010), beast (Drummond and Rambaut 2007), MrBayes (Ronquist et al. 2012b) Phylobayes (Lartillot et al. 2009), and DPPDiv (Heath et al. 2012). An important common feature of those new generation dating programs is that they accommodate the uncertainties in the fossil record to some extent. For example, unlike earlier dating analyses which assume that the ages of certain nodes on the phylogeny are known without error (Graur and Martin 2004), the new methods may use minimum- and maximum-age bounds to calibrate the molecular tree (e.g., Benton et al. 2009).

Because molecular sequence data provide information about distances only, but not about times and rates individually, this confounding effect between times and rates means that with uncertain calibrations, Bayesian estimation of times (and rates) will not converge to a point even if a huge amount of sequence data is analyzed. Yang and Rannala (2006) and Rannala and Yang (2007) developed the *infinite-sites theory*, which provides analytically the limiting posterior distribution when the number of sites in the sequence alignment approaches N→∞. Moreover, the prior will always exert an impact on the posterior, even in the analysis of large genome-scale data sets, and seemingly small differences between program implementations may translate to large differences in posterior time estimates (Inoue et al. 2010). Recently, dos Reis and Yang (2013) developed the *finite-sites theory*, which extends the infinite-sites theory of Yang and Rannala (2006) to the finite-sites case. Assuming the molecular clock, they were able to partition the uncertainty in posterior time estimates into two sources: That due to the uncertain fossil calibrations and that due to the limited sequence data (limited number of sites in the sequence alignment). Furthermore, when the sequence length N increases, the posterior variance in the time estimate approaches the infinite-data limit of Yang and Rannala (2006) at the rate 1/N.

The theory of dos Reis and Yang (2013) works for one single locus (site partition) and assumes the molecular clock. However, most modern molecular clock dating analyses are conducted under the relaxed clock models, as the strict clock is often violated, especially if the species are distantly related, and also use multiple loci (e.g., Bracken-Grissom et al. 2014; Christin et al. 2014). Thus, in this article, we consider the case of relaxed clock analyses of sequence data from multiple loci. Here the term locus refers to a site partition. The work will be an extension of the infinite-sites theory of Rannala and Yang (2007) to large but finite sequence data sets. We are interested in how the uncertainty in the posterior time estimates is reduced 1) when the number of sites at each locus N→∞, 2) when the number of loci L→∞, and 3) when both N and L→∞. The problem is not tractable analytically. Thus, we take the same strategy as in dos Reis and Yang (2013) and study a simple analogous case based on the normal distribution, and then use computer simulation and analysis of a real data set for a primate phylogeny to confirm predictions based on the analogy. We also use the primate data set to illustrate the application of the finite-sites theory we develop here to real data analysis.

Our theory applies to both the correlated-rate (Thorne et al. 1998; Rannala and Yang 2007) and independent-rate models (Drummond et al. 2006; Rannala and Yang 2007) for relaxing the molecular clock. In the analysis of sequence data from multiple loci (or site partitions), we assume the compound Dirichlet prior of dos Reis et al. (2014) for the locus rates (i.e., the rates appropriate for the loci). This prior is implemented in the mcmctree program. Most current Bayesian dating programs assume an independent and identical distribution of locus rates for multiple loci, which leads to overconfident posterior time estimates (dos Reis et al. 2014). The case of the i.i.d. prior for locus rates is discussed later.

## Theory and Methods

### Theoretical Analysis of a Normal Distribution Example

Following Rannala and Yang (2007) and dos Reis and Yang (2013), we consider a simple case of Bayesian estimation of confounded parameters involving the multivariate normal distribution. The example will help us to gain insights into the posterior uncertainty in the estimates of divergence times and substitution rates as the size of the sequence data set increases.

Suppose the data consist of a matrix $X=\left\{x_{ij}\right\}$, i = 1, 2, …, L; j = 1, 2, …, N; where each observation $x_{ij}$ is normally distributed. The model is

(1) $$x_{ij}=\mu_{\text{1}}+\mu_{\text{2}}+\xi_{i}+e_{ij},$$

where $e_{ij}\sim \text{N}(0,\sigma_{e}^{2})$ and $\xi_{i}\sim \text{N}(0,\sigma_{\xi }^{2})$, with both $\sigma_{e}^{2}$ and $\sigma_{\xi }^{2}$ given. This is a mixed linear model with $\mu_{\text{1}}$ and $\mu_{\text{2}}$ to be the fixed effects (parameters) and $\xi_{i}$ to be the random effects. However, the data or likelihood always depends on the sum $\mu_{\text{1}}+\mu_{\text{2}}$, but not on $\mu_{\text{1}}$ and $\mu_{\text{2}}$ separately, so that there is a problem of nonidentifiability in the construction of the model. We assign priors $\mu_{\text{1}}\sim \text{N}(-1,v_{\text{1}})$ and $\mu_{\text{2}}\sim \text{N}(1,v_{\text{2}})$ to estimate $\mu_{\text{1}}$ and $\mu_{\text{2}}$, with $v_{\text{1}}$ and $v_{\text{2}}$ known. We are interested in the posterior distribution (and in particular, the posterior means and variances) of $\mu_{\text{1}}$ and $\mu_{\text{2}}$ (and possibly of $\xi_{i})$ when the size of the data (N or L or both) is large.

Let ${\bar{x}}_{i}=\frac{1}{N}\sum_{j=1}^{N}x_{ij}$ and $\bar{x}=\frac{1}{LN}\sum_{i=1}^{L}\sum_{j=1}^{N}x_{ij}$ be the sample means. The likelihood is given by ${\bar{x}}_{i}\sim \text{N}(\mu_{\text{1}}+\mu_{\text{2}}+\xi_{i}$, $\sigma_{e}^{2}/N)$. The joint posterior density is

(2) $$\begin{array}{l} f(\mu_{1},\mu_{2},\{\xi_{i}\}|X)\propto f(\mu_{1})f(\mu_{2})\prod_{i=1}^{L}f(\xi_{i})f({\bar{x}}_{i}|\mu_{1},\mu_{2},\xi_{i})\\ =f(\mu_{1})f(\mu_{2})\prod_{i=1}^{L}\exp\left\{-\frac{\xi_{i}^{2}}{2\sigma_{\xi }^{2}}-\frac{{\left({\bar{x}}_{i}-\mu_{1}-\mu_{2}-\xi_{i}\right)}^{2}}{2\sigma_{e}^{2}/N}\right\}. \end{array}$$

This is a (L+2)-variant normal distribution. In the Appendix, we show that $\mu_{1}$ and $\mu_{2}$ have a bivariate normal posterior distribution with means, variances, and correlation to be

(3) $$\begin{array}{l} m_{1}=E(\mu_{1}|X)=-1+\frac{v_{1}\bar{x}}{v_{1}+v_{2}+v_{4}},\\ m_{2}=E(\mu_{2}|X)=1+\frac{v_{2}\bar{x}}{v_{1}+v_{2}+v_{4}},\\ s_{1}^{2}=V(\mu_{1}|X)=\frac{v_{1}(v_{2}+v_{4})}{v_{1}+v_{2}+v_{4}},\\ s_{2}^{2}=V(\mu_{2}|X)=\frac{v_{2}(v_{1}+v_{4})}{v_{1}+v_{2}+v_{4}},\\ \rho =\text{corr}(\mu_{1},\mu_{2}|X)=-\sqrt{1-\frac{v_{4}\left(v_{1}+v_{2}+v_{4}\right)}{\left(v_{1}+v_{4}\right)\left(v_{2}+v_{4}\right)}}, \end{array}$$

where $v_{\text{4}}=v_{\text{3}}/L$ and $v_{3}=\sigma_{e}^{2}/N+\sigma_{\xi }^{2}$.

The posterior variance of $\mu_{\text{1}}$ can be written as the sum of three terms

(4) $$s_{1}^{2}=v_{\infty }+\frac{1}{LN}a+\frac{1}{L}b,$$

where $\nu_{\infty }=\frac{v_{1}v_{2}}{v_{1}+v_{2}}$ is the infinite-data limit and where

(5) $$\begin{array}{l} a=\frac{\sigma_{e}^{2}v_{1}^{2}}{\left(\frac{\sigma_{\xi }^{2}}{L}+v_{1}+v_{2}\right)\left(\frac{\sigma_{e}^{2}}{LN}+\frac{\sigma_{\xi }^{2}}{L}+v_{1}+v_{2}\right)}\approx \sigma_{e}^{2}{\left(\frac{v_{1}}{v_{1}+v_{2}}\right)}^{2},\\ b=\frac{\sigma_{\xi }^{2}v_{1}^{2}}{\left(v_{1}+v_{2}\right)\left(\frac{\sigma_{\xi }^{2}}{L}+v_{1}+v_{2}\right)}\approx \sigma_{\xi }^{2}{\left(\frac{v_{1}}{v_{1}+v_{2}}\right)}^{2}. \end{array}$$

The approximations apply when L is large, which we assume here.

Based on equation (4), the following observations can be made. 1) When L is large and fixed, the posterior variance $s_{1}^{2}$ approaches the limit $v_{\infty }+b/L$ at the rate 1/N when N increases. 2) When N is fixed, $s_{1}^{2}$ approaches $\nu_{\infty }$ when L→∞, independently of N. The rate of approaching the limit is 1/L. The same limit is reached (a) when L→∞ with N fixed at a finite value and (b) when both N and L→∞.

#### Predictions for molecular clock dating

In Bayesian divergence time estimation under a relaxed clock model, we analyze sequence alignments at L loci, with N sites at each locus. The times (ages of nodes on the tree) are shared across all loci and are assigned a prior, often based on the birth–death process (e.g., Yang and Rannala 2006; Lepage et al. 2007). This prior also incorporates fossil calibrations, which come in the form of probability distributions. We assume that none of the node ages is known with certainty. When the data are analyzed, each locus i has an overall (mean) rate $\mu_{i}$, for i=1,2,…,L, and the $\mu_{i}$ are assigned a gamma-Dirichlet prior (dos Reis et al. 2014). The rates for branches or for nodes on the tree at the locus are then generated conditional on the locus rate $\mu_{i}$. Under the independent-rate model, the rates for branches in the tree at the locus are i.i.d. variables from the lognormal or gamma distribution with mean $\mu_{i}$ (Drummond et al. 2006; Rannala and Yang 2007). Under the correlated-rate model, $\mu_{i}$ is the rate at the root of the tree at the locus, from which rates for other nodes or branches evolve according to a stochastic process such as the geometric Brownian motion (Thorne et al. 1998; Thorne and Kishino 2002; Rannala and Yang 2007).

With this setup, the results for the normal distribution example (in particular, equation (4)) lead us to the following predictions concerning the uncertainty in the posterior estimates of divergence times. 1) When the number of loci L is large and fixed, the posterior variance of any node age has a nonzero limit when N→∞, and it approaches this limit at the rate 1/N. 2) When the number of sites at each locus N is large and fixed, the posterior variance of any node age has a nonzero limit when the number of loci L→∞, and it approaches this limit at the rate 1/L. Furthermore, the limiting variance for L→∞ is the same when N is fixed at different large values. The limiting variance when L→∞ reflects the uncertainties in the fossil calibrations, which cannot be reduced by further increase of the sequence data. This prediction suggests that to improve the precision of posterior time estimates, adding more loci (or site partitions) may be far more effective than increasing the length of sequence at each locus.

Using an analogy to equation (4), we may also partition the uncertainty in the posterior estimates of divergence times into three sources: The part due to the limited number of sites at each locus (that is, the term $\frac{a}{LN}$ in equation (4)), which disappears when N→∞, the part due to the limited number of loci (the term $\frac{b}{L}$ in equation (4)), which disappears when L→∞, and finally the part due to uncertainties in the fossil calibrations ($\nu_{\infty }$ in equation (4)), which cannot be reduced by further increase of sequence data. We illustrate this calculation in simulated and real data sets later.

### Design of the Simulation Experiment

We simulated sequence alignments on two phylogenies, with two or three species, respectively, to examine how the number of loci (L) and the sequence length at each locus (N) affect the uncertainty in posterior time estimates. For computational efficiency, we use the Jukes–Cantor (1969) model both to simulate data and to analyze them. Our focus in this study is the asymptotic behavior of posterior time estimation, and the precise nature of the assumed substitution model is unimportant (as long as the correct model is used). For example, if we use GTR+Γ (Tavaré 1986; Yang 1994a, 1994b) both to simulate and to analyze the data, the dynamics of posterior time estimation will be the same as under JC69, with parameters in the GTR+Γ model approaching the true values. Similarly, while we use small trees of only two or three sequences in the simulation (to reduce the computational cost) we expect the asymptotic dynamics of posterior time estimates to be independent of the dimension of the problem and to apply to larger trees with many species.

We describe the simulation for the three-species case and then comment on the two-species case. The true age of the root is fixed at $t_{\text{1}}=1$ and the age of the internal node is $t_{\text{2}}=0.5$. We consider one time unit to be 100 Myrs, so the two node ages are 100 and 50 Myrs, respectively. To simulate sequence alignments at L loci, we fix the overall rate at locus i at $\mu_{i}=0.1$ (meaning 0.1 substitutions per time unit or $10^{\text{-9}}$ substitutions per site per year). The rates for the four branches on the three-species tree are then generated as independent random variables from the lognormal distribution: $r_{ij}\sim \text{LN}({\text{log}\mu }_{i}-\sigma^{\text{2}}/2,\sigma^{\text{2}})$, for j=1,…,4 (Rannala and Yang 2007; equation (A.1)). We fix $\sigma^{\text{2}}=0.01$, which means that the molecular clock is slightly violated. Each branch length is then calculated by multiplying the time duration of the branch and the rate for the branch. Sequences at the locus are then simulated by evolving sequences along branches of the tree, using the evolver program in the paml package (Yang 2007). Simple R code is written to sample rates for branches and to generate the control files for evolver. The alignments for all L loci are then merged into one data file and constitute one data set, to be analyzed by the mcmctree program, also from the paml package. For the case of infinite sites (N=∞), branch lengths in the unrooted tree constitute the data. The number of replicates is 200.

In the two-species case, the true divergence time (or the age of the root) is t=1, and there are only two branches on the tree. All other settings are the same as in the case of three species. For example, the rates for the two branches are generated as independent lognormal variables with the mean $\mu_{i}=0.1$ and variance parameter $\sigma_{i}^{2}=0.01$.

The sequence data sets (each consisting of L alignments, each of N sites) are analyzed using mcmctree, whereas data sets of infinite sites (each consisting of L sets of branch lengths on the unrooted tree) are analyzed using the program Infinitesites. Both are Markov chain Monte Carlo (MCMC) programs from paml ver. 4.8. The age of the root is assigned the prior $t_{\text{1}}\sim \text{G}(100$, 100), with mean 1 and 95% interval (0.814, 1.205). This mimics the use of a soft-bound calibration at the root node of 81–121 Myrs. In the case of three species, the age of the internal node has a uniform prior between 0 and $t_{\text{1}}$, that is, $t_{\text{2}}|t_{\text{1}}\sim U(0,t_{\text{1}})$. The prior on $t_{\text{2}}$ is thus very diffuse, whereas the prior on $t_{\text{1}}$ is informative. This mimics the situation in which a fossil calibration is placed on $t_{\text{1}\;}$ but not on $t_{\text{2}}$. We assume a gamma-Dirichlet prior for rates at loci, $\{\mu_{i}\}\sim \text{gammaDir}(100$, 1000, 1) (dos Reis et al. 2014). The average rate over all loci is assigned a gamma prior, $\bar{\mu }=\frac{1}{L}\sum_{i=1}^{L}\mu_{i}\sim \text{G}(100$, 1000), with mean 0.1 and variance $10^{\text{-4}}$, and with the prior 95% interval 0.1±0.02 or $(0.08,0.12)\times 10^{\text{-8}}$ substitutions per site per year. Then the total rate for all loci, $L\bar{\mu }$, is partitioned into the locus rates using a uniform Dirichlet distribution (dos Reis et al. 2014). Given the locus rate $\mu_{i}$, the rates for branches have the i.i.d. prior from the lognormal distribution with mean $\mu_{i}$ and variance parameter $\sigma_{i}^{2}$. This is the so-called independent-rate model for relaxing the clock. A gamma-Dirichlet prior is assigned on parameters $\{\sigma_{i}^{2}\}\sim \text{gammaDir}(2$, 200, 1) as well: The mean across loci is assigned the gamma G(2, 200), whereas the total is partitioned among loci using a uniform Dirichlet.

In the MCMC analysis, the likelihood is calculated using Felsenstein's (1981) pruning algorithm. The length of the MCMC run is determined by running the program multiple times on the same data set to assess consistency between runs. Note that the amount of computation increases far more quickly with the increase of the number of loci than with the increase of the number of sites per locus. The analysis of each replicate data set (with L loci each of N sites) leads to a posterior sample of the divergence times, from which the posterior means and 95% equal-tail credibility intervals (CIs) are generated. Those are then averaged over the 200 replicates and reported.

### Estimation of Divergence Times on a Primate Phylogeny

We use the genomic data for six primate species of dos Reis and Yang (2013) to examine the uncertainty of posterior time estimates as a function of the loci used. The phylogeny with fossil calibrations is given later in Figure 3 The genomic data consist of six-species alignments of 9,992 protein coding genes. We removed ambiguous codons and alignment gaps and excluded genes with fewer than 200 codons, resulting in a data set of 7947 genes. We used only the third codon positions. Among those 7947 genes, the number of codons (or the number of third-position sites) ranged from 200 to 5055, with the median at 399.

To study the effect of the number of loci on the uncertainty of posterior time estimates, we sampled genes (third codon positions only) without replacement from the 7947 genes, to generate data sets with L=1, 5, 10, 20, 50, 100, 200, and 500 genes. For each L, 100 replicate data sets were generated. The data were then analyzed similarly to the simulated data, using mcmctree ver. 4.8. The sequence likelihood was calculated under the HKY$+\Gamma_{\text{5}}$ substitution model (Hasegawa et al. 1985; Yang 1994b). The prior of the divergence times was constructed using the calibrations of Figure 3 and the birth–death process, with the birth and death parameters $\lambda_{\text{BD}}=\mu_{\text{BD}}=1$, and sampling fraction $\rho_{\text{BD}}=0$. Those parameter values give a uniform kernel density (Yang and Rannala 2006, equation (5)). We used the gamma-Dirichlet prior gammaDir(2, 20, 1) for rates at loci ($\mu_{i})$: The average rate over all loci is assigned a gamma prior G(2, 20), with mean 0.1 and variance 0.005, whereas the total rate is partitioned among loci using a Dirichlet distribution (dos Reis et al. 2014). Here the time unit is 100 myr, so that the prior mean rate is $10^{\text{-9}}$ substitutions per site per year. Given the locus rate $\mu_{i}$, the rates for branches at the locus have the i.i.d. prior from the lognormal distribution with mean $\mu_{i}$ and variance parameter $\sigma_{i}^{2}$. Parameters $\sigma_{i}^{2}$ were assigned the prior gammaDir(2, 20, 1), to allow for the violation of the clock.

Note that in the normal distribution example N is the same for different i, whereas here N varies among loci. However, one may envisage an extra sampling step in which the number of sites N for each locus is sampled as a random variable from a common distribution and then the prediction based on the normal example should remain valid.

Besides the independent sampling scheme of generating data sets of L loci, we also used an alternative scheme, to partition the 7947 genes (or 3,982,327 third-codon-position sites) into L partitions. For each gene, we estimated the branch lengths by maximum likelihood, using RAxML (Stamatakis et al. 2012) and ranked the genes by tree length (sum of branch lengths). We then partitioned the genes into L=5, 10, 20, 50, 100, 200, and 500 partitions, with approximately the same number of genes in each partition, placing genes with similar tree lengths into the same partition. Here the tree length was used as a proxy for relative evolutionary rate. Note that the total number of sites analyzed (3,982,327) is always the same whatever the number of partitions L is. Each data set was analyzed exactly in the same way as above, with the L partitions treated as L independent loci. This way of analyzing the data is carried out here as it is common in molecular clock dating analysis, in which the genetic data are fixed and different partitioning strategies are evaluated (e.g., dos Reis et al. 2012). However, our predictions based on the normal distribution example are not expected to apply to this case.

## Results

### Simulation in the Case of Two Species

For the tree of two species, the true root age is t=1, and this is the only time parameter to be estimated. The posterior mean, the 95% CI, and the CI width (w) of time t are calculated for a number of values of L and N, with the results shown in Table 1, averaged over 200 simulated replicates. The posterior means are all very close to the true value. Here we focus on the uncertainty in the posterior time estimates, measured by $w^{\text{2}}$. Note that when the data set is large, $w^{\text{2}}$ should be approximately proportional to the posterior variance.

**Table 1.** Averages of posterior mean, 95% CI and CI widths (w) of divergence time (t) between two species

L	N	Mean	95% CI	w	($w/w_{\text{0}}{)}^{\text{2}}$, %	1 − ($w_{\infty }/w{)}^{\text{2}}$, %
0 (prior)	0	1.000	(0.814, 1.205)	0.392	100	50
1	10	1.000	(0.815, 1.204)	0.389	99	49
	$10^{\text{3}}$	1.002	(0.848, 1.171)	0.323	68	26
	$10^{\text{4}}$	1.001	(0.857, 1.161)	0.304	60	16
	∞	1.002	(0.858, 1.160)	0.302	60	15
2	10	1.004	(0.820,1.207)	0.387	98	48
	100	1.003	(0.834,1.188)	0.354	82	38
	$10^{\text{3}}$	1.001	(0.856,1.162)	0.306	61	17
	$10^{\text{4}}$	1.001	(0.861,1.155)	0.294	57	11
	∞	1.005	(0.866,1.158)	0.292	56	9
5	10	1.006	(0.825,1.205)	0.380	94	46
	100	1.006	(0.849,1.179)	0.330	71	29
	$10^{\text{3}}$	1.002	(0.864,1.155)	0.291	55	9
	$10^{\text{4}}$	1.004	(0.869,1.154)	0.285	53	5
	∞	1.003	(0.868,1.152)	0.284	53	4
10	10	1.010	(0.834, 1.205)	0.371	90	44
	$10^{\text{4}}$	1.003	(0.870, 1.151)	0.281	55	2
	∞	1.006	(0.872, 1.154)	0.282	53	3
100	10	1.026	(0.876,1.191)	0.315	90	22
	$10^{\text{4}}$	1.005	(0.873, 1.151)	0.278	51	0
	∞	1.005	(0.872, 1.151)	0.279	51	1
1000	10	1.043	(0.906, 1.194)	0.288	54	7
	$10^{\text{4}}$	1.004	(0.872, 1.150)	0.278	51	0
	∞	1.004	(0.872, 1.150)	0.278	51	0
10	100	1.003	(0.855, 1.166)	0.311	63	20
20		1.008	(0.866, 1.164)	0.298	58	13
50		1.009	(0.872, 1.159)	0.287	54	6
100		1.011	(0.877, 1.160)	0.283	52	4
200		1.010	(0.877, 1.158)	0.281	52	2
1000		1.010	(0.877, 1.156)	0.279	51	1
10	1000	1.005	(0.869, 1.155)	0.286	54	6
20		1.007	(0.873, 1.155)	0.282	52	3
50		1.005	(0.872, 1.152)	0.279	51	1
100		1.006	(0.873, 1.152)	0.279	51	1
200		1.005	(0.873, 1.151)	0.279	51	1
1000		1.004	(0.873, 1.150)	0.278	51	0

Notes: The true age is t = 1. Results for N=∞ are calculated using the infinite-sites theory of Rannala and Yang (2007). In the last two columns, ($w/w_{\text{0}}{)}^{\text{2}}$ is the ratio of the posterior to the prior variance, and 1 − ($w_{\infty }/w{)}^{\text{2}}$ is the percentage of the posterior variance that is due to limited sequence data. The results are averages over 200 replicate data sets.

**Figure 1.**
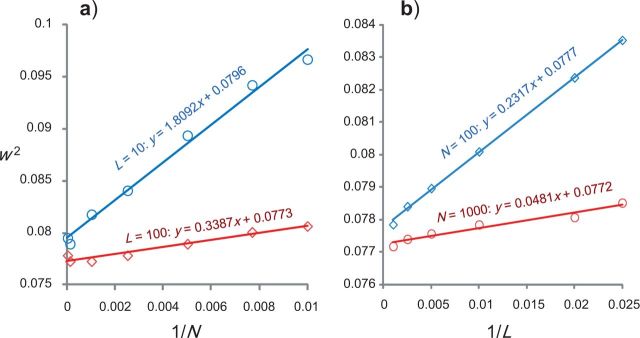
The finite-sites theory for two sequences. The square of the posterior 95% CI width ($w^{\text{2}}$) is plotted (a) against the reciprocal of the number of sites (1/N) in the alignment with the number of loci L fixed at 10 or 100, and (b) against the reciprocal of the number of loci (1/L) with the number of sites at each locus fixed at N=100 or N=1000.

In Figure 1a, we plot $w^{\text{2}}$ against 1/N with L fixed at either 10 or 100, and in Figure 1b, we plot $w^{\text{2}}$ against 1/L with N fixed at either 100 or 1000. According to our predictions based on the analogous normal distribution example, both plots should be linear when N and L are large, with positive (nonzero) intercepts. This is indeed the case.

In Figure 1a, the lines of best fit are $w^{\text{2}}=1.8092/N+0.0796$ for L=10 and $w^{\text{2}}=0.3387/N+0.0773$ for L=100. Both the slope and the intercept are smaller for the larger L, indicating more information in data of more loci.

In Figure 1b, the lines of best fit are $w^{\text{2}}=0.2317/L+0.0777$ for N=100, and $w^{\text{2}}=0.0481/L+0.0773$ for N=1000. The slope is smaller for the larger N (0.0418 for N=1000 compared with 0.2317 for N = 100). The intercepts for N = 100 and 1000 are nearly identical (0.0777 for N = 100 and 0.0773 for N = 1000). Based on the analogy with the normal distribution example, we expect the uncertainty to be the same for different N, when L→∞, so that those intercepts should be equal. Note that the intercept in the $w^{\text{2}}$ versus 1/L plot represents the limiting value when L→∞ and reflects the uncertainty in fossil calibrations and in the rate prior, uncertainty that cannot be reduced by increasing the amount of sequence data. We take $w^{\text{2}}$ = 0.0773 as the limiting uncertainty in time estimates for infinite sequence data. Before any molecular data are analyzed, $w^{\text{2}}$ = $0.391^{\text{2}}$ = 0.153, given by the prior, and this is reduced by a half (1 − 0.0773/0.153 = 0.495) when an infinite amount of sequence data is analyzed. The other half is due to the uncertainty in the prior and in the fossil calibration, and cannot be reduced by further increase of sequence data.

It is noteworthy that the posterior uncertainty does not approach zero when the amount of sequence data increases without bound (that is, when N→∞, when L→∞ or when both N and L→∞) (Table 1). From the intercepts of the $w^{\text{2}}$ versus 1/L plots in Figure 1b and from the results of Table 1, we estimate the limiting posterior 95% CI to be (0.871, 1.148), with w = 0.278 or $w^{\text{2}}=0.0773$. In other words, even with an infinite amount of sequence data, the 95% CI (87.1–114.8Ma) spans 28 Myrs. Compared with the prior, which has the 95% CI width to be 39.1 Myrs (Table 1), the posterior interval at the infinite-data limit is 30% narrower, and the posterior variance is ~50% smaller.

It is also interesting to examine the posterior uncertainty when the data set is small, before the asymptotics are reliable. With only L = 2 loci, increasing the sequence length N from 100 to 1000 reduces the interval width from 0.354 to 0.306. This is a reduction of 14% (= 1 − 0.306/0.354) in the CI width or a reduction of 25% (= 1 − $0.306^{\text{2}}/0.354^{\text{2}})$ in the posterior variance. However, increasing N further to $10^{\text{4}}$ reduces the posterior CI width to 0.294, with much less effect. This trend of diminishing returns is because very quickly most of the posterior variance is due to the uncertain fossil calibrations, which cannot be reduced by adding sequence data. Increasing the sequence length has even less effect when a larger number of loci is used. It seems that with L≤ 5 loci analyzed, $10^{\text{4}}$ sites per locus are nearly as good as an infinite number of sites, and with L≥10 loci analyzed, 200 or 1000 sites are not much worse than an infinite number of sites.

### Simulation in the Case of Three Species

In the tree for three species, there are two node ages, with the true ages to be $t_{\text{1}}=1$ and $t_{\text{2}}=0.5$. The posterior means, the 95% CIs, and the CI widths for $t_{\text{1}}$ and $t_{\text{2}}$ are shown in Table 2, averaged over 200 replicates. Again, the posterior means are close to the true values, so we focus on the posterior uncertainty measured by $w^{\text{2}}$.

**Table 2.** Posterior means, 95% CIs and CI widths of divergence times ($t_{\text{1}}$ and $t_{\text{2}}$) for three species

		$t_{\text{1}}$					$t_{\text{2}}$				
L	N	Mean	95% CI	w	($w/w_{\text{0}}{)}^{\text{2}}$, %	1 − ($w_{\infty }/w{)}^{\text{2}}$, %	Mean	95% CI	w	($w/w_{\text{0}}{)}^{\;\text{2}}$,%	1 − ($w_{\infty }/w{)}^{\text{2}}$, %
0	0	1.000	(0.814, 1.205)	0.392	100	50	0.500	(0.025, 1.026)	1.001	100	98
10	20	1.005	(0.839 , 1.188)	0.349	79	37	0.509	(0.307, 0.758)	0.451	20	91
	50	1.010	(0.855, 1.179)	0.324	68	26	0.509	(0.366, 0.678)	0.312	10	80
	200	1.006	(0.865, 1.160)	0.295	56	11	0.505	(0.411, 0.612)	0.201	4.0	52
	∞	1.003	(0.870, 1.151)	0.281	51	2	0.502	(0.433, 0.579)	0.146	2.1	9
100	10	1.023	(0.876, 1.186)	0.310	62	20	0.506	(0.394, 0.636)	0.242	5.8	67
	20	1.015	(0.874, 1.171)	0.296	57	12	0.504	(0.412, 0.609)	0.197	3.9	50
	50	1.010	(0.875, 1.161)	0.286	53	6	0.505	(0.427, 0.592)	0.165	2.7	29
	200	1.005	(0.872, 1.152)	0.280	51	1	0.503	(0.434, 0.580)	0.146	2.1	9
	∞	1.005	(0.873, 1.151)	0.278	50	0	0.503	(0.436, 0.576)	0.140	2.0	1
10	100	1.004	(0.858, 1.165)	0.307	61	18	0.506	(0.393, 0.637)	0.244	5.9	68
20		1.002	(0.862, 1.156)	0.294	56	11	0.506	(0.413, 0.613)	0.200	4.0	52
50		1.006	(0.871, 1.156)	0.286	53	6	0.503	(0.425, 0.591)	0.166	2.8	30
100		1.007	(0.873, 1.155)	0.282	51	3	0.502	(0.430, 0.582)	0.152	2.3	16
200		1.008	(0.875, 1.155)	0.280	51	1	0.502	(0.433, 0.578)	0.146	2.1	9
400		1.007	(0.874, 1.153)	0.279	50	1	0.501	(0.433, 0.575)	0.142	2.0	4
1	1000	0.997	(0.847,1.163)	0.315	64	22	0.506	(0.380,0.655)	0.275	7.5	74
2		1.002	(0.858,1.159)	0.301	59	15	0.505	(0.404,0.622)	0.218	4.7	59
5		1.003	(0.866,1.154)	0.288	54	7	0.501	(0.420,0594)	0.174	3.0	36
10		1.002	(0.867, 1.150)	0.283	52	4	0.503	(0.429, 0.587)	0.158	2.5	23
20		1.005	(0.872, 1.152)	0.281	51	2	0.501	(0.431, 0.579)	0.148	2.2	12
50		1.004	(0.872, 1.151)	0.279	50	1	0.502	(0.434, 0.577)	0.143	2.0	6
100		1.004	(0.872, 1.151)	0.279	50	1	0.502	(0.435, 0.576)	0.141	2.0	3
200		1.004	(0.872, 1.150)	0.278	50	0	0.502	(0.435, 0.575)	0.140	2.0	1
400		1.004	(0.872, 1.150)	0.278	50	0	0.502	(0.436, 0.575)	0.140	2.0	1

Notes: The true node ages are $t_{\text{1}}=1$ and $t_{\text{2}}=0.5$. Note that the limiting CI width for $t_{\text{2}}$ is $w_{\infty }=0.139=\sqrt{0.0193}$ (Fig. 2). See legend to Table 1.

**Figure 2.**
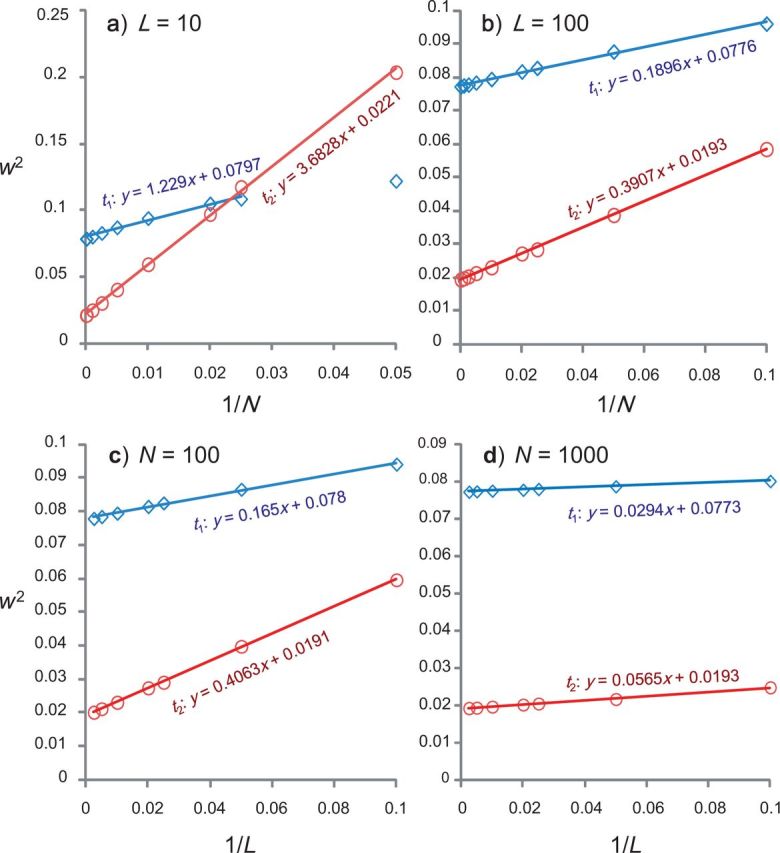
The finite-sites theory for three sequences. The square of the posterior 95% CI widths ($w^{\text{2}}$) for the two divergence times ($t_{1}$ and $t_{2}$) is plotted against 1/N, with the number of loci L fixed at 10 a) or 100 b); and against 1/L, with the number of sites fixed at N=100 c) or 1000 d). In (a) for L=10, the point N=20 is not used to fit the line for $t_{\text{1}}$ because those values of L and N appear too small for the linear asymptotic trend to apply.

We plot $w^{\text{2}}$ against 1/N with L fixed at 10 or 100 in Figure 2a and b, and against 1/L with N fixed at 100 or 1000 in Figure 2c and d. Both kinds of plots show near perfect linear relationships when N and L are large, confirming our predictions based on the normal distribution example. The asymptotic linear relationship holds well for N≥40 and for L≥10.

For both $t_{\text{1}}$ and $t_{\text{2}}$, the intercepts in the plots of $w^{\text{2}}$ against 1/L are nearly identical for N = 100 and N = 1000 (Fig. 2c, d). For example, the intercept for $t_{\text{1}}$ is 0.0780 at N = 100 and 0.0773 at N = 1000, whereas for $t_{\text{2}}$ it is 0.0191 at N = 100 and 0.0193 at N = 1000. Those results confirm our prediction that the limiting posterior uncertainty when L→∞ does not depend on N. The minor differences may be attributed to small sampling errors due to limited number of replicates and to the fact that our L and N values are not very large. The results suggest that with an infinite amount of sequence data, the limiting values are $w_{1}^{2}\approx 0.0773=0.278^{\text{2}}$ for $t_{\text{1}}$ and $w_{2}^{2}\approx 0.0193=0.139^{\text{2}}$ for $t_{\text{2}}$, with $w_{\text{1}}/w_{\text{2}}$ = 2 = $t_{\text{1}}/t_{\text{2}}$. The one-dimensional limiting posterior is almost entirely dominated by the prior for $t_{\text{1}}$.

We also examined posterior uncertainties in small data sets before the asymptotic theory is reliable. Note that the prior and data information for $t_{\text{1}}$ are nearly the same as in the simulation for two species. The posterior uncertainty for $t_{\text{1}}$ is also very similar to and slightly smaller than that for the two-species case. The reduced interval or improved precision is due to the information coming from the third sequence. At the infinite-data limit, the posterior for $t_{\text{1}}$ is exactly the same as in the two-species case, with the posterior CI to be (0.871, 1.148), and w = 0.278 or $w^{\text{2}}$ = 0.0773. Overall the pattern for $t_{\text{1}}$ is the same as in the two-species case.

The results for $t_{\text{2}}$ are very different from those for $t_{\text{1}}$. There is much weaker information in the prior about $t_{\text{2}}$ than about $t_{\text{1}}$. As a result, when molecular data are analyzed, the uncertainty in $t_{\text{2}}$ is reduced far more dramatically than that for $t_{\text{1}}$. Increase of both the sequence length N and the number of loci L leads to reduction in the posterior uncertainty of $t_{\text{2}}$, and improvement is seen even at large values of L or N (Table 2). Even so, the percentage of posterior uncertainty that is due to sequence data goes down quickly. For example, for the sequence length N=1000, this percentage is 59, 36, and 6% for L=2, 5, and 50 loci, respectively. Furthermore, we note that the linear prediction becomes reliable for $t_{\text{2}}$ earlier than for $t_{\text{1}}$; in other words, the linear relationship applies for smaller values of N and L for $t_{\text{2}}$ than for $t_{\text{1}}$.

### Estimation of Divergence Times on a Primate Phylogeny

**Figure 3.**
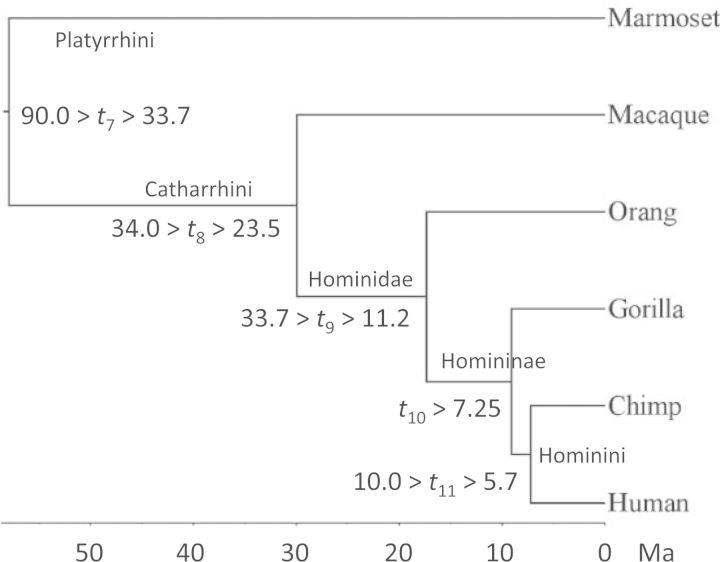
Phylogeny of six primate species. Fossil calibrations are shown next to the nodes on the tree. The joint bounds (for nodes 7, 8, 9, and 11) are implemented as soft uniform bounds with a sharp minimum (1% of tail probability on the left) and a soft maximum (5% of tail probability on the right) (Yang and Rannala 2006, figure 2). The minimum bound (on node 10) is implemented using the truncated Cauchy distribution (Inoue et al. 2010).

The posterior means, the 95% CIs, and the CI widths (w) of divergence times on the primate phylogeny of Figure 3 ($t_{\text{7}}$–$t_{\text{11}})$ are shown in upper panel of Table 3, when L loci are sampled at random from the 7949 genes are analyzed. Here a gene or locus means all the third codon positions of the protein coding gene. The prior is shown as well (L = 0 in Table 3).

**Table 3.** Posterior means, 95% CIs and CI widths of divergence times for the primate data set

	$t_{\text{7}}$			$t_{\text{8}}$			$t_{\text{9}}$			$t_{\text{10}}$			$t_{\text{11}}$		
L	Mean	95%CI	$w_{\text{7}}$	Mean	95%CI	$w_{\text{8}}$	Mean	95%CI	$w_{\text{9}}$	Mean	95%CI	$w_{\text{10}}$	Mean	95%CI	$w_{\text{11}}$
0 (prior)	0.632	(0.348, 0.921)	0.573	0.297	(0.238, 0.346)	0.108	0.216	(0.120, 0.318)	0.198	0.129	(0.076, 0.233)	0.157	0.077	(0.058, 0.101)	0.043
Independent loci sampled from 7947 genes^a^															
1	0.651	(0.408, 0.899)	0.491	0.304	(0.243, 0.347)	0.104	0.199	(0.131, 0.280)	0.149	0.105	(0.075, 0.158)	0.083	0.075	(0.058, 0.099)	0.041
5	0.667	(0.501, 0.844)	0.343	0.314	(0.257, 0.350)	0.093	0.185	(0.140, 0.234)	0.094	0.091	(0.074, 0.118)	0.044	0.07	(0.058, 0.090)	0.032
10	0.666	(0.527, 0.815)	0.288	0.319	(0.267, 0.352)	0.085	0.183	(0.146, 0.222)	0.076	0.089	(0.074, 0.110)	0.036	0.067	(0.057, 0.083)	0.026
20	0.645	(0.541, 0.753)	0.212	0.324	(0.280, 0.353)	0.073	0.179	(0.151, 0.208)	0.057	0.084	(0.073, 0.099)	0.026	0.064	(0.057, 0.076)	0.019
50	0.652	(0.573, 0.728)	0.155	0.327	(0.291, 0.353)	0.062	0.177	(0.156, 0.198)	0.042	0.082	(0.074, 0.092)	0.018	0.062	(0.057, 0.070)	0.013
100	0.652	(0.588, 0.714)	0.126	0.329	(0.299, 0.353)	0.054	0.179	(0.162, 0.196)	0.034	0.08	(0.074, 0.089)	0.015	0.061	(0.057, 0.068)	0.011
200	0.65	(0.597, 0.703)	0.106	0.33	(0.304, 0.353)	0.049	0.178	(0.163, 0.192)	0.029	0.08	(0.074, 0.087)	0.013	0.06	(0.057, 0.066)	0.009
500	0.648	(0.601, 0.696)	0.095	0.33	(0.306, 0.352)	0.046	0.178	(0.165, 0.191)	0.026	0.08	(0.074, 0.086)	0.012	0.06	(0.057, 0.065)	0.008
Partitions of 7947 genes^b^															
1	0.675	(0.485, 0.881)	0.396	0.313	(0.255, 0.349)	0.094	0.185	(0.144, 0.230)	0.086	0.089	(0.074, 0.113)	0.039	0.068	(0.057, 0.088)	0.031
5	0.647	(0.553, 0.740)	0.187	0.319	(0.279, 0.350)	0.071	0.185	(0.160, 0.222)	0.062	0.084	(0.074, 0.094)	0.020	0.064	(0.057, 0.073)	0.016
10	0.664	(0.583, 0.741)	0.158	0.319	(0.282, 0.349)	0.067	0.182	(0.161, 0.201)	0.040	0.083	(0.074, 0.093)	0.018	0.064	(0.057, 0.071)	0.014
20	0.662	(0.588, 0.732)	0.144	0.319	(0.285, 0.349)	0.064	0.182	(0.163, 0.200)	0.038	0.083	(0.074, 0.092)	0.017	0.063	(0.057, 0.070)	0.013
50	0.660	(0.592, 0.724)	0.132	0.320	(0.288, 0.349)	0.061	0.182	(0.164, 0.199)	0.035	0.083	(0.075, 0.090)	0.016	0.063	(0.057, 0.069)	0.012
100	0.657	(0.594, 0.718)	0.124	0.321	(0.291, 0.350)	0.059	0.181	(0.164, 0.197)	0.034	0.082	(0.075, 0.090)	0.015	0.063	(0.057, 0.068)	0.012
200	0.655	(0.597, 0.713)	0.116	0.322	(0.294, 0.350)	0.056	0.180	(0.164, 0.196)	0.032	0.082	(0.075, 0.089)	0.014	0.062	(0.057, 0.068)	0.011
500	0.652	(0.599, 0.706)	0.107	0.324	(0.298, 0.350)	0.053	0.179	(0.165, 0.194)	0.029	0.081	(0.075, 0.088)	0.013	0.062	(0.057, 0.067)	0.010

^a^A random sample of L genes (out of the 7947) are analyzed. The results are averages over 100 replicates. ^b^The 7947 genes are partitioned into L partitions by rate.

**Figure 4.**
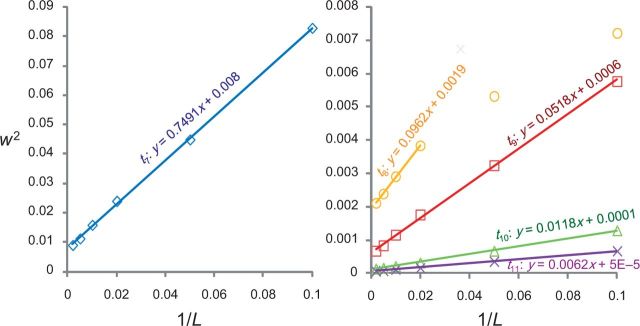
The finite-sites theory applied to the analysis of genomic sequence data from six primate species (Fig. 3). The square of the posterior 95% CI widths ($w^{\text{2}}$) for the 5 node ages ($t_{\text{7}}$, $t_{\text{8}}$, $t_{\text{9}}$, $t_{\text{10}}$, and $t_{\text{11}})$ is plotted against the reciprocal of the number of loci, sampled at random from 7947 protein coding genes (with only the third codon positions used).

In Figure 4 we plot the posterior uncertainty (measured by $w^{\text{2}})$ for the five divergence times in the phylogeny ($t_{\text{7}}$–$t_{\text{11}})$ against 1/L. In all cases except $t_{\text{8}}$, $w^{\text{2}}$ shows a strong linear relationship with 1/L as long as L≥ 10, consistent with our predictions. For $t_{\text{8}}$, the linear relationship holds well only for much larger values of L, that is, only if L≥50. For small L before the asymptotics become reliable, the posterior CI width is smaller than the predicted value from the straight line (see plot for $t_{\text{8}}$ in Fig. 4). As in the simulation for three species, the asymptotic theory starts to become reliable for smaller values of L if the node has a less informative prior calibration. dos Reis and Yang (2013) suggested the use of the 95% prior interval width divided by the prior mean as a measure of prior or calibration precision. This is 0.91, 0.37, 0.90, 1.02, and 0.56 for $t_{\text{7}}$, $t_{\text{8}}$, $t_{\text{9}}$, $t_{\text{10}}$, and $t_{\text{11}}$, respectively, with $t_{\text{8}}$ having the most precise calibration.

**Figure 5.**
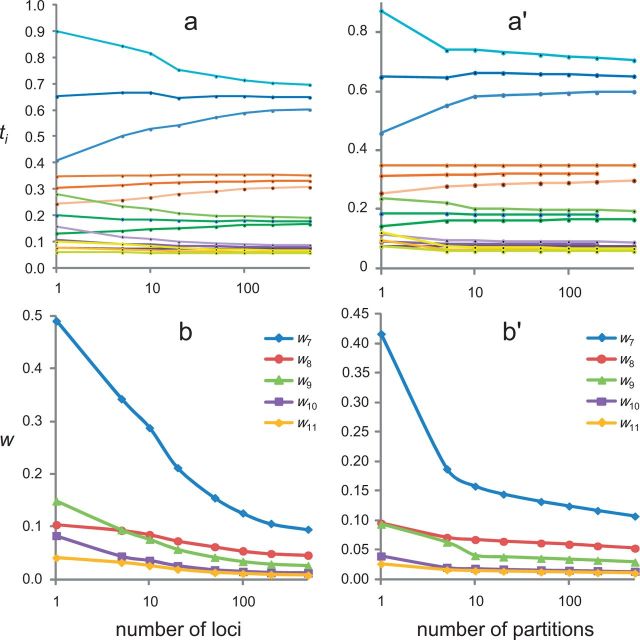
Posterior distribution of divergence times in the primate tree of six species. In (a)-(b), the data consist of L independent loci, sampled at random from the 7947 genes ( upper panel of Table 3) The results are averages over 100 replicates. In (a′)-(b′), the 7947 genes are grouped into L partitions according to the relative rate of substitution. In (a) and (a′) the posterior mean and 95% CI for each node age $t_{i}$ are plotted. In (b) and (b′), the 95% CI width (w) is plotted.

The intercept of the regression line, which is an estimate of the limiting uncertainty ($w^{\text{2}})$ with an infinite amount of sequence data, is 0.0080, 0.0019, 0.0006, 0.0001, and 0.00005, for the five nodes $t_{\text{7}}$, $t_{\text{8}}$, $t_{\text{9}}$, $t_{\text{10}}$, and $t_{\text{11}}$, respectively. These are very close to the $w^{\text{2}}$ values for L = 500 in upper panel of Table 3: 0.0090, 0.0021, 0.00068, 0.00014, 0.000064 for $t_{\text{7}}$–$t_{\text{11}}$, indicating that 500 loci may be close to an infinite amount of sequence data.

We then consider the alternative strategy of partitioning the 7947 loci (third codon positions) into L site partitions. In this case, the same total number of sites are always used in the analysis whatever L is. The results are summarized in lower panel of Table 3. Note that our asymptotic theory based on the normal distribution example does not apply to this case, as the setups of the statistical estimation problems are very different. The case of L=1 (lower panel of Table 3) corresponds to the concatenation analysis, in which all sites are analyzed as one partition, with the same substitution model and the same set of branch rates for all sites. This is still commonly used in molecular dating analyses (e.g., Christin et al. 2014). The posterior CIs from this concatenation analysis are slightly wider than those in the independent sampling scheme for L=5 loci for $t_{\text{7}}$ and $t_{\text{8}}$, and are slightly smaller for $t_{\text{9}}$–$t_{\text{11}}$ (upper panel of Table 3), but they are larger for all node ages than in the independent sampling scheme for L=10 loci, even though 10 independent loci constitute only about 0.13% (= 10/7947) of the data used in the concatenation analysis.

At the other end, when L = 500 loci, the posterior 95% CI widths are similar between the independent sampling scheme (upper panel of Table 3) and the partition analysis (lower panel of Table 3), even though the former used only about 6.3% (= 500/7947) of the data as in the latter. Indeed the independent sampling scheme had very slightly smaller CI widths for $t_{\text{7}}$–$t_{\text{11}}$.

Those results suggest that to increase the precision of posterior time estimates, it is far more effective to increase the number of loci than to increase the sequence length at each locus, at least when the sequence length at each locus is not too small (in our case, N≥200 at every locus).

## Discussion

### Unconventional Nature of the Estimation Problem

The confounding effect of times and rates makes Bayesian estimation of species divergence times using uncertain fossil calibrations an unconventional estimation problem. Here we highlight two important differences. First, in a conventional Bayesian estimation problem, the estimate (posterior mean, say) will converge to the true parameter value when the size of the data set (N) increases without bound, with the posterior variance decreasing to zero. In large data sets, the posterior variance is typically proportional to 1/N. The Bayesian method of parameter estimation is known to be statistically consistent. However, such convergence to truth does not occur in Bayesian divergence time estimation, when the amount of sequence data increases without bound and when the fossil calibrations involve uncertainties and are fixed. The model is not fully identifiable, and the posterior variance will not approach zero even if an infinite amount of sequence data is analyzed (Britton 2005; Yang and Rannala 2006). Second, in a conventional Bayesian estimation problem, the impact of the prior will become unimportant when the data size increases, and in large data sets, the posterior will be dominated by the data or likelihood. This is not the case when we use genetic sequence data to estimate absolute times and absolute rates. Even with an infinite amount of sequence data, the posterior will remain sensitive to the prior on times (which includes the calibration information) and the prior on rates, as highlighted in the infinite-sites theory (Yang and Rannala 2006; Rannala and Yang 2007).

In this study, we characterized the posterior uncertainty, measured by the posterior variance or the square of the posterior CI width ($w^{\text{2}})$, in Bayesian relaxed clock dating analysis using sequence data from multiple loci. We used an analogous normal distribution example to make *qualitative* predictions about the posterior variance of times when the number of loci L and the sequence length N are large but finite. The predictions are then confirmed in specific cases using computer simulation and analysis of a primate data set. The posterior variances of divergence times have three components. The first is due to sampling errors in the branch lengths due to the limited sequence length N. If L is large, this component goes to zero at the rate 1/N. The second component is due to evolutionary rate variation among loci and among branches at each locus according to the relaxed clock model. This decreases to zero in proportion to 1/L. The third component is due to uncertainty in fossil calibrations. This cannot be reduced by increasing the amount of sequence data. For most phylogenetic analysis, the locus is not very short (with N>1000 sites, say). Then use of multiple loci (or site partitions) will be the most effective approach to improving the precision of posterior time estimation under relaxed clock models.

The importance of analyzing multiple loci or site partitions to reduce posterior uncertainty does not seem to be well appreciated in the literature. Many dating analyses commonly use the concatenation method, by which multiple genes are concatenated into one “supergene,” with one set of rates for branches used in the model (e.g., Christin et al. 2014). Also empirical biologists appear to be surprised by the lack of big improvement in estimation precision with the addition of molecular data (e.g., Mulcahy et al. 2012), even though this is expected from theory (Yang and Rannala 2006; Rannala and Yang 2007). Given the persistent uncertainty in the posterior even when large genome-scale data sets are analyzed, the posterior means or medians of divergence times may not represent the posterior distribution well. Thus, we suggest that in molecular dating analyses, posterior CIs or standard deviations of divergence times be reported in addition to the posterior means or medians.

### Assumptions and Validity of the Theory

Our theory is constructed not through mathematical proofs but by statistical intuition through an analogy with a toy example that is analytically tractable. However, there are a number of differences between the toy example and the divergence time estimation problem. Here we discuss or speculate on which of those differences matter to our qualitative results. First, the toy example assumes an additive model (equation (1)), but the model in the Bayesian dating problem is multiplicative (i.e., a branch length or sequence distance is the product of time and rate). However, we consider this difference to be unimportant. If we take the exponential on both sides of equation (1), the additive model will become a multiplicative one, with

(6) $$X_{ij}=M_{\text{1}}\cdot M_{\text{2}}\cdot \Xi_{i}\cdot E_{ij},$$

where $X_{ij}=e^{x_{ij}}$, $M_{\text{1}}=e^{\mu_{1}}$, and so on. By assuming that the random effect $\Xi_{i}$ and the error $E_{ij}$ have lognormal distributions and by assigning lognormal priors on parameters $M_{\text{1}}$ and $M_{\text{2}}$, equation (6) specifies exactly the same model as equation (1), so that exactly the same results will be obtained in the Bayesian analysis. Furthermore, in large data sets, the variances for a parameter x and for its function y=y(x) are approximately related as var$(y)=\text{var}(x)\times |\frac{\text{d}y}{\text{d}x}{|}^{2}$, where the derivative is evaluated at the true parameter values and is a constant scale factor. Thus, if var(x) is proportional to 1/N, so is var(y). Such transforms or reparametrizations typically affect the sample size needed for the asymptotics to work well but not the asymptotic behavior itself. In other words, var(x) may become proportional to 1/N sooner (for smaller N) than var(y), but with sufficiently large N, both var(x) and var(y) should be proportional to 1/N. Similarly we do not consider the distributional forms (normal in the toy example vs. multinomial for sequence alignments in the dating problem) to be important.

In the simulation we fixed the locus rate $\mu_{i}$ to be constant among loci (equal to the prior mean). We suggest that the asymptotic behavior should be the same if one samples $\mu_{i}$ from the prior instead: In both cases, the data are independent among loci given the parameters of interest ($\mu_{\text{1}}$ and $\mu_{\text{2}}$ in the toy example vs. times and the average rate across loci in the dating problem). Another assumption we made both in the model and in the simulation is that the species phylogeny and species divergence times are shared among all loci. A number of biological processes can cause the gene trees at individual loci to differ from the species tree. For example, the coalescent process in ancestral species may cause the gene tree topology to differ from the species tree and also the gene divergences to be older than the species divergences. This source of uncertainty is ignored here. As a result, our theory may apply only to distantly related species so that the coalescent times are negligible compared with the species divergence times.

Although many factors affect divergence time estimation, we consider two of them to be particularly important to the qualitative results of this article: The fossil calibrations and the prior model for variable rates among loci and among branches. Here we discuss the first factor and treat the second below in the next subsection.

We have assumed that the fossil calibrations are uncertain (i.e., they are not fixed constants) but correct. We have ignored the challenges of summarizing the fossil evidence to generate uniform bounds or other statistical distributions for use as fossil calibrations in the dating analysis. Erroneous calibrations may have major effects on divergence time estimation. In some situations, they may produce extremely precise but grossly wrong time estimates whereas in others the conflicts between fossils and between fossils and molecules may lead to multimodal posteriors. The impact of incorrect calibrations on the asymptotic behavior of divergence time estimation is not well understood (dos Reis and Yang 2013). Also our theory assumes that the fossil calibrations used in the dating analysis are fixed, and does not apply if more and more fossil calibrations are added into the dating analysis. We speculate that our asymptotic results will also apply to the joint analysis of morphological characters and molecular sequence data, as conducted by Ronquist et al. (2012a), or in the joint analysis of fossil occurrence data and molecular sequence data, as in (Wilkinson et al. 2011; see also Bracken-Grissom et al. 2014), if the morphological data or fossil occurrence data are fixed, whereas the amount of molecular data increases. Further research is needed to test those predictions.

### Prior Models for Substitution Rates Among Loci

In this study, we have assumed the compound Dirichlet prior of dos Reis et al. (2014) for the locus rates (averages rates for the loci). Most current Bayesian dating programs implement the i.i.d. prior for locus rates, assuming that the rates for loci have an independent and identical distribution. Suppose $\mu_{i}$ has a distribution with variance v. With L loci, the average of $\mu_{i}$ over all L loci, $\bar{\mu }=\frac{1}{L}\sum_{i=1}^{L}\mu_{i}$, will have the variance v/L. For large L, this variance will be very small. In analysis of large molecular data sets, the posterior of times and rates is nearly one dimensional (e.g., dos Reis and Yang 2013, figure 3), and the near certain knowledge of one parameter, such as the age of a single node on the tree or the average rate over all loci, will be sufficient to resolve the nonidentifiability problem and make all parameters be estimated with near certainty. This high precision is an artefact of the unreasonable prior. One consequence of this i.i.d. prior is that if the rate prior is mis-specified (with a wrong prior mean, say), the posterior time estimates may be grossly wrong and yet very precise (e.g., dos Reis et al. 2014, figure 1).

Here we mention two priors that avoid this problem. The gamma-Dirichlet prior implemented by dos Reis et al. (2014) assigns a gamma distribution for the average rate across all loci: $\bar{\mu }=\frac{1}{L}\sum_{i=1}^{L}\mu_{i}\sim \text{G}(\alpha_{\mu }$, $\beta_{\mu })$, and then partitions the total rate for all L loci ($L\bar{\mu })$ using a Dirichlet distribution with concentration parameter α. The means, variance, and correlation are given as

(7) $$\begin{array}{l} \begin{array}{l} E(\mu_{i})=\frac{\alpha_{\mu }}{\beta_{\mu }}, \end{array}\\ var(\mu_{i})=\frac{\alpha_{\mu }}{\beta_{\mu }^{2}}\left[1+\frac{\alpha_{\mu }+1}{L\alpha +1}(L-1)\right]\to \frac{\alpha_{\mu }}{\beta_{\mu }^{2}}\left[1+\frac{\alpha_{\mu }+1}{\alpha })\right],\;\text{if}\;L\to \infty ,\\ \rho =\text{corr}(\mu_{i},\mu_{j})=\frac{L\alpha -\alpha_{\mu }}{L(\alpha +1)+(L-1)\alpha_{\mu }}\to \frac{\alpha }{\alpha +1+\alpha_{\mu }},\;\text{if}\;L\to \infty \end{array}$$

(dos Reis et al. 2014; equations (6–7)).

The second prior may be referred to as the conditional i.i.d. prior, and is not yet implemented. We assume that the rate $\mu_{i}$ for locus i has the distribution $\mu_{i}|\bar{\mu }\sim \text{G}(\alpha ,\alpha /\bar{\mu })$, where α is the shape parameter and measures how variable the rates are among loci, and $\bar{\mu }$ is the mean of the prior distribution. Then we assign a hyperprior $\bar{\mu }\sim \text{G}(\alpha_{\mu },\beta_{\mu })$, with shape parameter $\alpha_{\mu }$ and $\beta_{\mu }$. Under this prior, we have

(8) $$\begin{array}{l} \begin{array}{l} E(\mu_{i})=E(E(\mu_{i}|\bar{\mu }))=\frac{\alpha_{\mu }}{\beta_{\mu }}, \end{array}\\ var(\mu_{i})=E(V(\mu_{i}|\bar{\mu }))+V(E(\mu_{i}|\bar{\mu }))=E(\frac{{\bar{\mu }}^{2}}{\alpha })+V\left(\bar{\mu }\right)\\ =\frac{1}{\alpha }\left(\frac{\alpha_{\mu }}{\beta_{\mu }^{2}}+\frac{\alpha_{\mu }^{2}}{\beta_{\mu }^{2}}\right)+\frac{\alpha_{\mu }}{\beta_{\mu }^{2}}=\frac{\alpha_{\mu }}{\beta_{\mu }^{2}}\left[1+\frac{1+\alpha_{\mu }}{\alpha }\right],\\ \rho =\text{corr}(\mu_{i},\mu_{j})=\frac{\alpha }{\alpha +1+\alpha_{\mu }}. \end{array}$$

It does not seem possible to integrate out $\bar{\mu }$ to generate the joint prior distribution $f(\mu_{\text{1}}$, $\mu_{\text{2}}$, … , $\mu_{L}|\alpha_{\mu }$, $\beta_{\mu }$, α) analytically. However, one may use MCMC to integrate over $\bar{\mu }$.

A drawback of the gamma-Dirichlet prior is that it depends on the size of the data (the number of loci L), but it has the advantage that the joint prior distribution $f(\mu_{\text{1}}$, $\mu_{\text{2}},\ldots ,\mu_{L}|\alpha_{\mu }$, $\beta_{\mu }$, α) is tractable analytically (dos Reis et al. 2014, equation (3)). The conditional i.i.d. prior appears to match the toy example and our simulation more closely than the gamma-Dirichlet prior. Nevertheless, the two priors are extremely similar for large L, with the same means, variances, and correlations. They appear to have the same asymptotic dynamics.

One aspect of the prior for rates that may be unrealistic biologically is the assumption that given the rates for loci ($\mu_{\text{1}},\ldots ,\mu_{L})$, the rates for branches are independent among loci. In other words, the different loci will provide independent realizations of the rate-drift process. Analysis of real data has highlighted the existence of both the gene effect, which is described by the prior model $f(\mu_{i}|\bar{\mu })$, and the lineage effect of substitution rates (Ho 2014). The lineage effect here means that some branches on the tree have high rates in all or most loci whereas some other branches have low rates. Current prior models ignore such rate correlation among loci, and may be expected to exaggerate the information content in the sequence data (Thorne et al. 1998). An even more extreme case may be as follows. Imagine one has five genes but analyzes them as 10 partitions, separating each gene into two arbitrary partitions. Such an analysis will generate more precise time estimates, but the high precision is spurious, because the two halves of the same gene may have very similar evolutionary rate trajectories and do not constitute two independent realizations of the rate-drift process.

## Funding

This work was supported by a grant from the Biotechnological and Biological Sciences Research Council (BB/J009709/1) to Z.Y., and grants from Natural Science Foundation of China (31301093, 11301294 and 11201224) to T.Z.

## Appendix

To obtain the joint posterior distribution of $\mu_{\text{1}}$ and $\mu_{\text{2}}$, we integrate out $\xi_{i}$ in equation (1). The exponent in equation (1) is

(A.1) $$\begin{array}{l} -\left(\frac{1}{2\sigma_{\xi }^{2}}+\frac{1}{2\sigma_{e}^{2}/N}\right)\xi_{i}^{2}+\frac{2N}{2\sigma_{e}^{2}}\left({\bar{x}}_{i}-\mu_{1}-\mu_{2}\right)\xi_{i}\;\\ -\frac{N}{2\sigma_{e}^{2}}{\left({\bar{x}}_{i}-\mu_{1}-\mu_{2}\right)}^{2}\\ =-\frac{\sigma_{e}^{2}+N\sigma_{\xi }^{2}}{2\sigma_{e}^{2}\sigma_{\xi }^{2}}\left(\xi_{i}-\frac{N\sigma_{\xi }^{2}}{\sigma_{e}^{2}+N\sigma_{\xi }^{2}}\left({\bar{x}}_{i}-\mu_{1}-\mu_{2}\right)\right)\\ -\frac{1}{2\left(\sigma_{e}^{2}/N+\sigma_{\xi }^{2}\right)}{\left({\bar{x}}_{i}-\mu_{1}-\mu_{2}\right)}^{2}. \end{array}$$

Let $\sigma_{e}^{2}/N+\sigma_{\xi }^{2}=v_{3}$. Thus we have

(A.2) $$\begin{array}{l} f(\mu_{1},\mu_{2}|X)\propto f(\mu_{1})f(\mu_{2})\exp\left\{-\sum_{i}\frac{1}{2v_{3}}{\left({\bar{x}}_{i}-\mu_{1}-\mu_{2}\right)}^{2}\right\}\\ \propto \exp\{-\frac{1}{2v_{1}}{\left(\mu_{1}+1\right)}^{2}-\frac{1}{2v_{2}}{\left(\mu_{2}-1\right)}^{2}\\ -\sum_{i}\frac{1}{2v_{3}}{\left({\bar{x}}_{i}-\mu_{1}-\mu_{2}\right)}^{2}\}. \end{array}$$

This is a bivariate normal density with standard form

(A.3) $$\begin{array}{l} f(\mu_{1},\mu_{2}|X)=\frac{1}{2\pi s_{1}s_{2}\sqrt{1-\rho^{2}}}\exp\{-\frac{1}{2(1-\rho^{2})}({\left(\frac{\mu_{1}-m_{1}}{s_{1}}\right)}^{2}\;\\ -2\rho \frac{\mu_{1}-m_{1}}{s_{1}}\frac{\mu_{2}-m_{2}}{s_{2}}+{\left(\frac{\mu_{2}-m_{2}}{s_{2}}\right)}^{2})\}. \end{array}$$

The means, variances, and correlation could be obtained by equating the coefficients of $\mu_{1}^{2}$, $\mu_{2}^{2}$, $\mu_{\text{1}}\mu_{\text{2}}$, $\mu_{\text{1}}$, and $\mu_{\text{2}}$ in the exponent in equation (A.2) to those in equation (A.3). Here it seems simpler to integrate out $\mu_{\text{2}}$ to get the marginal distribution of $\mu_{\text{1}}$, which is univariate normal. Completing the square for $\mu_{\text{2}}$ in the exponent of equation (A.2), we have

(A.4)

where const. is independent of $\mu_{\text{1}}$ and $\mu_{\text{2}}$. Thus after $\mu_{\text{2}}$ is integrated out, the terms involving $\mu_{\text{1}}$ in the exponent of equation (A.2) becomes

(A.5)

Thus $m_{\text{1}}$ and $s_{\text{1}}$ are as given in equation (2). Similarly one obtains $m_{\text{2}}$ and $s_{\text{2}}$. By equating the coefficient of $\mu_{1}^{2}$ in the exponents of equations (A.2) and (A.3), we have ($v_{\text{1}}+v_{\text{4}})$/(2$v_{\text{1}}v_{\text{4}})=1/(2s_{1}^{2}(1-\rho^{2}))$, and by noting the sign of the coefficient of $\mu_{\text{1}}\mu_{\text{2}}$ in equation (A.2), we obtain ρ as in equation (2).
